# Emergency pharmacy workforce views and experience related to the provision of pharmaceutical care during mass gathering events: the FIFA World Cup Qatar 2022™ experience

**DOI:** 10.3389/fpubh.2023.1286637

**Published:** 2023-12-08

**Authors:** Lina Naseralallah, Nour Isleem, Shimaa Aboelbaha, Abdulrouf Pallivalapila, Shaikha Alnaimi, Moza Al Hail

**Affiliations:** ^1^Department of Pharmacy, Hamad Medical Corporation, Doha, Qatar; ^2^School of Pharmacy, Institute of Clinical Sciences, College of Medical and Dental Sciences, Sir Robert Aitken Institute for Medical Research, University of Birmingham, Edgbaston, Birmingham, United Kingdom; ^3^College of Pharmacy, QU Health, Qatar University, Doha, Qatar

**Keywords:** mass gathering, FIFA World Cup, pharmacist, emergency disaster, disaster risk reduction

## Abstract

**Purpose:**

This study aimed to explore emergency pharmacy workforce perspectives and experiences in providing pharmaceutical care during mass gathering events (i.e., FIFA World Cup Qatar 2022™).

**Methods:**

A qualitative methodology was employed using focus groups discussions. Emergency pharmacists across Hamad Medical Corporation were invited to participate using a combination of purposive and snowball sampling. Focus groups were audio-recorded, transcribed verbatim, and validated. Inductive thematic analysis was undertaken to generate key themes and subthemes.

**Results:**

Four focus groups were conducted which included 21 participants and generated five major themes. Whilst participants had mixed opinions in relation to their preparedness to practice during the World Cup, they perceived their experience as successful and smooth. The primary perceived facilitators were management support, mobile medical units, and high public health awareness. The main highlighted barriers were related to staff insufficiency, medications availability, and cultural and language challenges. Participants recommended pharmacist’s role identification in mass gatherings, development of pharmacy action plan, and offering simulation training and pharmacy-specific training.

**Conclusion:**

Despite the perceived barriers, pharmacists reported positive views in relation to their experience in providing pharmaceutical care during mass gatherings. Future research should focus on the development of theory-driven action framework for pharmacy departments to adopt during mass gatherings.

## Introduction

Mass gathering is defined by the World Health Organization (WHO) as an event with more than 1,000 person assembled at a particular location for a specific purpose and for a defined period of time (1). Mass gathering events pose considerable public health challenges to health authorities and governments. This could be attributed to the substantial health risks and pressure that accompany such events with the potential for a surge in demand for healthcare services that might strain the functioning of the host country’s health system (2). Additionally, previous research showed that such events increase the workload especially for emergency medical services (3–6). It may also cause delayed emergency responses via various mechanisms and stretch the resources of the local emergency services beyond their capacity (7). Nonetheless, these events may present opportunities for long-term positive impacts, including stronger public health systems following the events, or residents and visitors that are better informed about how to protect themselves from certain diseases (8, 9).

The World Cup is the largest global sport event that was hosted by the State of Qatar in 2022. It was estimated that approximately 1.4 million fans from around the world visited the country between 20 November and 18 December to attend this event (10). This large number of attendees had the potential to impose a hefty burden on public health and response capabilities. This situation is particularly dire in Qatar given its relatively small size and that the number of visitors accounted for nearly half of the country’s population. (11). Additionally, Qatar has a desert climate with a temperature ranging between 57°F (13.8°C) and 107°F (42°C) (12). Previous events conducted in countries with similar climates (e.g., Hajj in Mecca, Saudi Arabia) showed that there could be an increased risk of heat exhaustion and sun stroke (13). Moreover, hosting such events during the coronavirus disease 2019 (COVID-19) era raises concerns about the spread of the pandemic in addition to other infectious diseases (14).

Therefore, preparing the healthcare system, particularly the emergency medical services, and implementing a strategic action plan was an imperative component of Qatar’s preparation for the World Cup. This was to ensure timely and effective healthcare provision to visitors and residents. This is also part of the health security pillar of the country’s Healthy 2022 World Cup project in partnership with the WHO, FIFA and Qatar Supreme Committee for Delivery and Legacy (15). Hamad Medical Corporation (HMC) is the principal public nonprofit healthcare provider in Qatar. It launched many strategies to prepare the medical staff and health facilities for functioning during the World Cup (15). As part of HMC preparedness efforts, the pharmacy department has implemented many initiatives to prepare its workforce particularly emergency department (ED) pharmacists (16).

Pharmacy workforce is a crucial part of the healthcare system. In recent years, the scope of practice for pharmacists has been broadened to undertake expanded patient-oriented activities such as promoting public health and performing frontline roles, which could help in relieving pressure on other areas of the healthcare system. The latter was particularly evident amidst the COVID-19 pandemic when pharmacists promptly adapted new roles and effectively contributed to health sector response (17). Previous research highlighted the importance of having a pharmacy-based strategic plan during mass gathering events (13, 18, 19). Nevertheless, a scoping literature search revealed that, although action plans exist for other departments or for healthcare systems in general, less attention has been directed at preparation measures specific to pharmacy departments (13, 18, 19). Furthermore, other findings from existing literature showed that pharmacists demonstrated an unsatisfactory level of preparedness for emergency disasters (20, 21). Although previous research has looked into the preparedness of the pharmacy workforce for emergency disasters (22–25), there is a dearth of studies on pharmacists’ preparedness for mass gathering events. A recently published paper from Qatar showed that although emergency pharmacists had a high level of awareness in regard to emergency disasters that could occur during the World Cup, their level of readiness was still moderate (26). It is therefore imperative to investigate possible underpinnings and planning gaps and to capture pharmacists’ reflections on their experiences and roles during the World Cup.

This study aimed to explore emergency pharmacy workforce views and experiences regarding their preparedness and potential roles during the World Cup. It also examined the barriers encountered and learnings for the future. Findings from our research could provide guidance for policymakers in Qatar and worldwide when planning preparedness measures and strategic care programs for future mass gathering events.

## Methods

### Study design

A descriptive qualitative approach using face-to-face focus groups was used in this study. Focus groups were selected as the method of choice, primarily to stimulate and encourage interaction and in-depth discussions between participants which could gauge a wide range of ideas and experiences, and explore various perspectives arising from debates within groups. They are also useful in assessing the needs and identifying enablers, challenges, and making recommendations for improvements and future plans (27, 28). The Consolidated Criteria for Reporting Qualitative Research (COREQ) was followed in the reporting of this study (29).

### Ethical considerations

The study obtained ethical approval from the Medical Research Center (MRC) at HMC in 2022 (MRC-0122-680). Prior to the commencement of the focus groups, all participants provided written signed consent.

### Sampling

Eligible participants were current pharmacists, clinical pharmacists, and pharmacists in administrative positions working in the ED across HMC. A combination of purposive sampling and snowball sampling was undertaken for the recruitment of participants. Recruitment continued until data saturation was achieved based on the number of emergent themes and following discussions among the research team (30). An email describing the nature and purpose of the study was sent to the candidates with the research information sheet attached. Participants were allowed to withdraw from the study at any point up to one-week post-focus group.

### Data collection

A semi-structured discussion guide comprising 13 questions was designed based on the responses from the first stage of this project (26), existing literature, research team expertise, and through iterations and feedback within the research team. The topic guide pilot was tested on two pharmacists working in the ED. Minor amendments were made post-piloting. The interview guide consisted of four parts: (1) the views of the pharmacy workforce on their level of preparedness and their experiences during the World Cup; (2) an overview of any training received and previous experience in relation to mass gatherings followed by more focused questions on the World Cup; (3) facilitators and barriers to care provision during the World Cup; and (4) lessons learned and recommendations for future mass gatherings.

The face-to-face focus groups took place within HMC premises. A researcher (LN or NI) moderated the discussion with the assistance of an observer (LN or NI), who helped in facilitation, when needed, and took field notes. Ample opportunities were given to explore further points raised by participants. Both researchers are registered clinical pharmacists (Doctor of Pharmacy (PharmD) holders) practicing in HMC with experience in moderating focus group discussions. Each focus group comprised of either pharmacists and clinical pharmacists or pharmacy supervisors. This was to ensure that the composition of the group has a dynamic that allows flow of content, stimulates conversation, and increases the speed of information generation. It also helped to avoid the impact of power differentials which could inhibit participants from expressing their thoughts freely (31).

The sessions were audio-recorded. All audio recordings were transcribed verbatim by a researcher (SA). Subsequently, two authors independently (LN or NI and SA) verified the transcripts for accuracy. Data were stored securely on a password protected HMC server and personal identifiable information was removed from transcripts immediately following each focus group.

### Data analysis

Data were analyzed by inductive thematic analysis using a six-step framework as a guide (32). This involved repeated familiarization with the transcripts, identifying initial codes, generating themes, reviewing themes, defining key themes and sub-themes, and finally producing the report. The principal researcher (LN) read through each transcript line-by-line several times to identify and arrange prominent codes and impactful quotes according to statements made by the participants. These codes were then organized using NVivo software, version 12 (QSR International). A second investigator (SA) reviewed both codes and raw transcripts thus strengthening the validation of study results. All authors met to discuss the coding, similarities and differences until consensus was reached on the key themes and subthemes.

## Results

### Demographics

Between January and March 2023, four focus groups were conducted, with three of them involving pharmacists or clinical pharmacists and one with pharmacists in administrative positions. Due to the limited number of supervisors/directors responsible for ED operations, additional focus groups with this population were not feasible. The total number of participants was 21, with 4 to 6 participants per session. The average duration of the focus groups was 53 min, ranging from 49 to 58 min. As illustrated in Table 1, majority of participants were males (62%), between 30–40 years of age (52.4%), and had a range of 5–15 years of experience in HMC (52.4%).

**Table 1:** Participants’ demographic and practice characteristics.

Characteristics	*N* (%)
Gender Male Female	13 (61.9) 8 (38.1)
Age <30 30–40 41–50	7 (33.3) 11 (52.4) 3 (14.3)
Position Pharmacists Clinical pharmacist Clinical pharmacist specialists Supervisors	6 (28.5) 7 (33.3) 3 (14.2) 5 (23.8)
Years of practicing in HMC <5 5–15 16–25 >25	4 (19.0) 11 (52.4) 4 (19) 2 (9.5)

HMC, Hamad Medical Corporation; *N*, number; %, percentage.

### Major overarching themes

**Figure 1:**
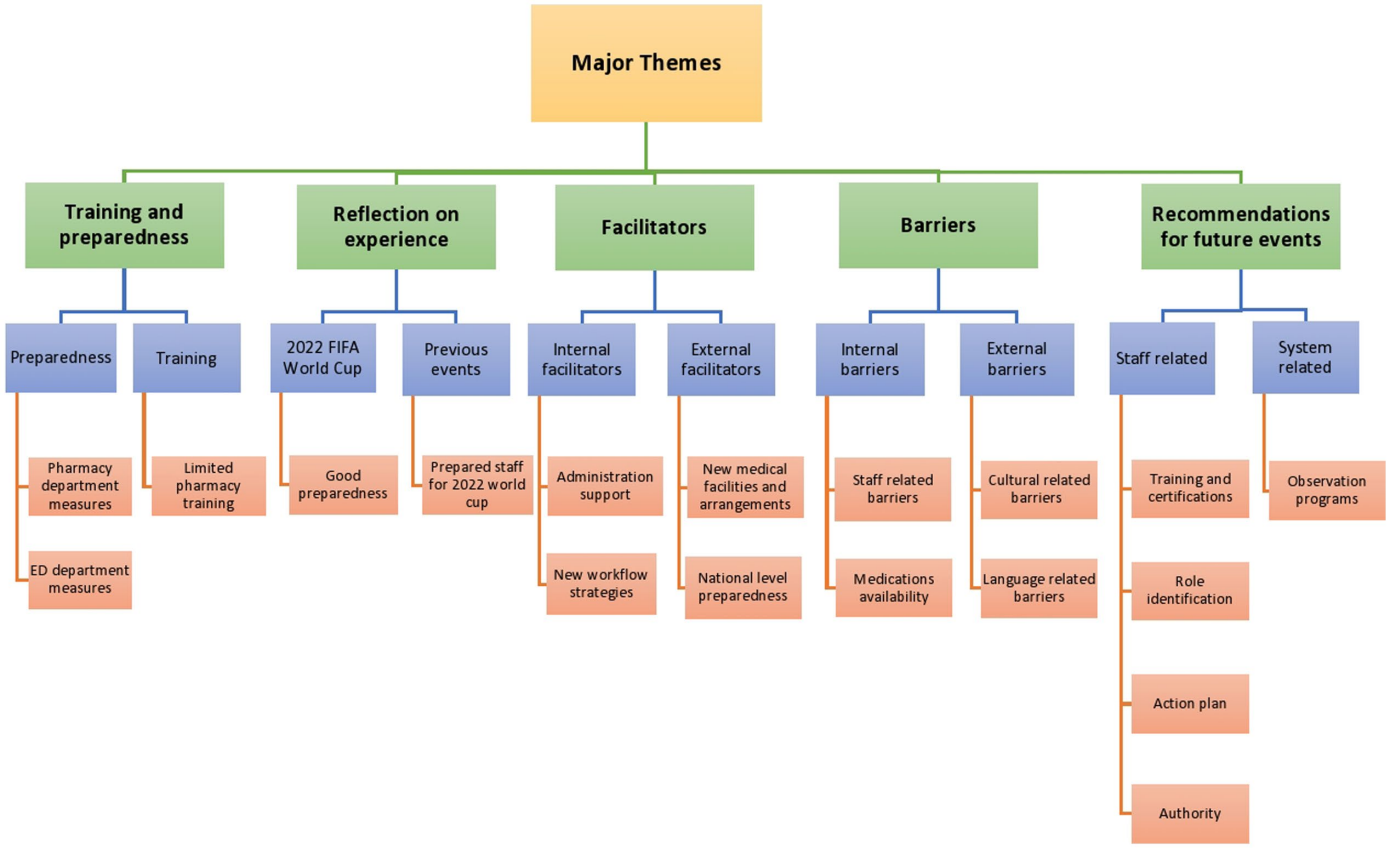
Conceptual map.

Five major themes emerged from the focus groups: preparedness and training; facilitators; barriers; reflection on the experience of mass gathering events; and recommendations for future mass gathering events. These themes were further subdivided into categories and subcategories. Figure 1 and Table 2 summarize information on the generated themes and subthemes.

**Table 2:** Major themes and subthemes emerged during the focus groups.

Theme	Subtheme	Summary of findings
Training and Preparedness	1.a Preparedness 1.b Training	Participants’ training prior to the FIFA World Cup as well as the measures taken to prepare the ED pharmacy staff for the championship were explored. Overall participants reported having a variety of measures implemented including translation machines as well as pre-established protocols and pathways providing instructions for employees at the time of disasters. They have also reported receiving general basic training but there was no ED pharmacy specific training prior to the FIFA WC.
Reflection on the experience of mass gathering events	2.a FIFA World Cup experience 2.b Previous experiences	This theme emerged from respondents’ reflection on their experience during the World Cup as well as previous mass gatherings they experienced while working in the ED. There was an overall perception that the ED pharmacists and supervisors were well prepared for the event and that previous mass gatherings have impacted their preparedness positively
Facilitators	3.a Internal facilitators 3.b External facilitators	This theme represents participants’ opinions on factors that facilitated their workflow during the World Cup. A total of two subthemes were identified, internal and external facilitators. Internal facilitators refer to those relating to HMC while external facilitators were any other factors that are independent of HMC and positively affected the workflow of pharmacist within the ED. An example of internal and external facilitators were administration support and national level preparedness, respectively
Barriers	4.a Internal barriers 4.b External barriers	Under this theme, participants’ opinions on factors that hindered their workflow during the FIFA World Cup were explored. Responses were categorized into two subthemes, internal and external barriers. Internal barriers refer to those relating to HMC while external barriers were any other factors that are independent of HMC and adversely affected the workflow of pharmacist within the ED. An example of internal barrier was insufficient staff and external barrier was cultural challenges
Recommendations for future mass gathering events	5.a Pharmacy staff related recommendations 5.b System related recommendations	Participants suggested a variety of recommendations based on their experience that should be considered by ED and pharmacy department administrations for future mass gathering events held in Qatar or in any other country around the globe. Majority of recommendations were related to pharmacy trainings and having observation programs sent to other mass gathering events to learn from their successes and failures

## 1 Theme 1: preparedness and training for the FIFA world cup

### 1.1 Preparedness measures implemented to facilitate the workflow during the FIFA world cup

Measures were classified into pharmacy department-related measures which refer to those implemented by the pharmacy administration to pharmacists operating within the ED; and ED department-related measures which refer to those implemented by the ED for all medical professions working within the department.

#### 1.1.1 Pharmacy department-related measures

Regarding pharmacy department-specific measures, participants agreed that preparatory strategies involved two aspects, ED staff and ED medication.

For staff measures, participants reported that the number of shifts for clinical pharmacists increased during the World Cup to ensure clinical pharmacy services were available around-the-clock. This was achieved mainly through the deployment of staff.

“We had expansion in the usual working hours, it was only covering 16 h seven days a week, but when the World Cup kicked in, we expanded to 24/7. Also, we changed areas of coverage, for some staff during that 1 month. Moreover, we made sure that if there was an emergency, the staff would be ready to come at any time. Memo was disseminated to ensure this.” (FC1, P2).

Regarding ED medications measures, participants reported that the formulary in HMC was sufficient to address major needs. However, medication stocks were increased, and specific antidotes were added.

*“Actually, the number of medications on the ED list before ten years was around 200 items, but now we have 600 items. This was not only for the World Cup purposes, but mainly because in the last few years Qatar hosted many mass gatherings. So, this was a key modification to improve the readiness of the facility to meet such needs.” (FC4, P3).*

*“The formulary was sufficient; we did not add any medications. I think we only added two antidotes.” (FC4, P2).*

#### 1.1.2 ED-related measures

The ED had pre-established protocols and pathways to ensure a prompt and effective response in case of emergencies. The protocols, for example, contain instructions on how to triage cases, categorize patients according to their severity of illness, label medications, and how to fill and handle medication trolleys that can be used in disasters.

*“Before the World Cup, all ED staff were prepared for disasters by having specific protocols and guidelines to respond to the event in a very short time. F*or example, the way the pharmacists should label the medications during downtime and how to triage patients if pharmacists have any mass casualties *(FC4, P4).*

*“We implemented what we call pharmacy on wheel. It is a service that brings medications to different locations, often using a vehicle or mobile unit. This concept is particularly useful during major incidents. So, pharmacy on wheels means that necessary medications would be mobilized from the ED pharmacy to the gate from which patient will be admitted” (FC4, P2).*

Participants mentioned that one of the important measures implemented in the ED was directed at addressing language barriers that may have arisen from the diversity of population visiting Qatar during the World Cup. It involved having a language bank and portable translation machines. Additionally, staff speaking different languages were also available to translate when necessary.

*“We were expecting to receive visitors from different nationalities. So, we prepared a language bank which is a hotline that the staff can use to contact trained translators with a medical background. In case the language was not available in the bank, the staff had another alternative which is translation machines or Google Translate. Also, we have staff from all around the world who were able to help in translating in some scenarios” (FC3, P5).*

### 1.2 Training offered prior to the FIFA world cup

Participants stated that they received hospital-accredited general training to be able to act appropriately during any major incidents. This training is required for all HMC staff on a regular basis (annually or every two years). Additionally, the ED undertook practice drills and offered general training for all medical professions including pharmacists. However, the participants highlighted the lack of pharmacy-specific training prior to the World Cup.

*“During the preparation, we did not receive any training specific to pharmacists, but our staff in the ED here were part of the practice drills and some general training that was offered by the ED. We also had to take the Intermediate Life Support course as a part of the license renewal requirements every two years. Also, we receive annual training, such as the fire and safety course, which is a general course too.” (FC2, P2).*

## 2 Theme 2: reflection on the experience of providing care during mass gathering events

### 2.1 Reflection on the FIFA world cup experience

Most pharmacists expressed positive perceptions of their work experience during the championship and felt that they were well-prepared. They referred to the fact that this preparedness was something that developed over the years and not just in the period leading up to the event. Furthermore, they described their workflow during the event as being smooth and under control.

*“From my point of view, we were prepared, and to some extent, everything was almost clear in terms of preparedness measures and pathways, whether from the pharmacy itself or amongst us as pharmacists in the ED.” (FC1, P6).*

Although some pharmacists and clinical pharmacists regarded their experience as successful and that they had good awareness level, they reported that their preparedness level was inadequate given the challenges they faced such as lack of training and the extra workload.

*“No we were not fully prepared. However, I honestly think we were aware of what to expect, but not how to practically manage certain scenario” (FC1, P3).*

From the pharmacy supervisor’s perspective, they indicated that they were satisfied with the preparedness level of their staff as well as their own preparedness.

*“Yes, of course. I think the staff was prepared enough for any emergency disaster in general. It is important to highlight that we were prepared for the worst-case scenarios, but the event was too smooth.” (FC4, P4).*

### 2.2 Reflection on previous mass gathering events

Participants were asked about their previous experience with mass gathering events hosted by Qatar and the impact of these experiences on their level of preparedness for the World Cup. They confirmed that they have attended previous championships such as the Asian Games and Arab Cup, and that these events had positive influence on their preparedness for the World Cup. The majority of participants reported that attending these events gave them insight into the logistics, organization, and practical aspects of managing a large-scale event, which enabled them to be better prepared for this one.

*“Yes, I attended and participated in the Asian Cup training, and I joined the ED team to help prepare for it. I believe that this experience gave me training, prepared me for causality about major incidents, and helped me understand what to expect from the current World Cup.” (FC2, P5).*

## 3 Theme 3: facilitators to providing care during the FIFA world cup

### 3.1 Internal facilitators

#### 3.1.1 Administration support

Participants emphasized the importance of the pharmacy administration support prior and during the event. The management of work shifts, deployment of staff, and the oversight of the creation of ED medication lists were all listed as examples of this support.

*“A key enabler for the workflow was leadership support. It involved moving the staff around and preparing them to handle any major crisis.” (FC3, P1).*

#### 3.1.2 New workflow strategies in the ED

Patients were divided into two groups when they visited the ED during the event which are residents and visitors. Haya card holders (visitors) had a specific pathway and were typically permitted to receive medication for 7 days only. This was done to control drug supplies and to make sure that HMC could provide high-quality care to all patients.

“The work was carried out ordinarily for residents. The list of medications that our directors here at ED produced before the World Cup was distributed to Haya card holders, some of which were provided free of charge, while others had a discounted price, and they were permitted to obtain a quantity that was adequate for up to 7 days only.” (FC2, P5).

### 3.2 External facilitators

#### 3.2.1 New medical facilities and arrangements

Participants highlighted the Ministry of Public Health’s efforts to implement new external medical facilities and services as an external facilitator. These facilities included sobering and sexual assault centers and mobile command units in stadiums and fan zones. The mobile clinics were managed by dedicated crews in order to ensure these facilities were run smoothly. These facilities contributed to reducing the workload on ED as reported by participants.

*“In order to handle* var*ious medical issues and lessen the pressure on ED in hospitals, they built things like mobile clinics in the fan zones and stadiums. Our ED became more like a tertiary setting” (FC1, P1).*

*“New clinics opened during the World Cup, such as the sexual abuse and sobering centers which were part of the facilitators that reduced the load on us.” (FC1, P3).*

#### 3.2.2 National level preparedness

On the national level, two facilitators were reported by participants. First, shifting the championship from summer to winter, which significantly decreased the number of heat-related illness cases visiting the ED and consequently decreased the workload on the department. Second, the government’s efforts to raise public knowledge of the dangers of overcrowding by outlining the steps that must be taken to stop the spread of COVID-19 and other infectious diseases. Additionally, the Ministry of Public Health launched campaigns for health promotion and public education.

*“We were already prepared by changing the time of the World Cup from summer to winter which reduced the expected number of heat-related illness cases” (FC1, P4).*

*“I guess the media campaigns, the news, and the announcements were already preparing the public and the entire nation. So, it seemed like everyone was getting ready. As a result, of course, it gives you the impression that you are already supported” (FC1, P2).*

## 4 Theme 4: barriers to providing care during the FIFA world cup

### 4.1 Internal barriers

#### 4.1.1 Staff-related barriers

The lack of pharmacy-specific training was mentioned as one of the main barriers that may have affected the level of preparedness.

*“Unfortunately, the pharmacy did not offer training prior to the World Cup.” (FC1, P4).*

*“I feel we are missing pharmacy specific accredited courses that could prepare the pharmacists for any disaster” (FC1, P3).*

#### 4.1.2 Medications availability

Participants mentioned that during the World Cup they faced issues related to medications availability. For instance, some patients were on medications, doses or formulations in their home countries that were not available in Qatar. While participants emphasized that medication stocks were adequate, they still faced some unprecedented challenges as a result of global medication shortages.

*“The big challenge was those patients who came from their home country with medications, doses, or formulations that are not available in HMC. Not only that, but they are also not available in Qatar, and some of these medications, to be honest with you, are essential medications such as monoclonal antibodies that they are on for life.” (FC1, P6).*

*“Some medications were out of stock but that was due to the global shortage of these medications, which everybody knows about.” (FC4, P2).*

### 4.2 External barriers

#### 4.2.1 Culture-related factors

In Qatar, alcohol consumption and having premarital relationships are strictly regulated. This was especially concerning during the World Cup as the country welcomed visitors from all around the globe with different cultural backgrounds. Thus, pharmacists expressed that they had to navigate cultural and ethical challenges while dealing with alcoholism and sexually transmitted diseases.

*“Foreign visitors have no problem with drinking as it is in their culture. However, it is against the law here. Because they are not here on a permanent basis, you cannot technically discharge them into a rehabilitation center. So, we struggled with how to discharge these patients.” (FC2, P4).*

#### 4.2.2 Language-related factors

Although a language bank and translation machines were available during the championship, participants indicated that they still encountered difficulties communicating with patients who spoke different, less common languages and emphasized the need for interpreters in addition to these measures.

*“Actually, one of the challenges was language. So, we brought special machines into the ED, but in my opinion, this was not enough because, in some cases, if you need to understand others in more detail, you need an interpreter.” (FC3, P2).*

## 5 Theme 5: recommendations for future mass gathering events

### 5.1 Pharmacy staff-related recommendations

#### 5.1.1 Training and certifications

Participants suggested various recommendations to improve pharmacy training prior to mass gathering events. These recommendations mainly encompassed providing pharmacy-specific training, making it continuous and mandatory, and involving all pharmacists (i.e., not only ED staff). It should also include simulations of real-life disaster scenarios. Other suggestions entailed that pharmacists practicing in ED should be board certified in emergency medicine pharmacy. Additionally, participants highlighted the importance of improving pharmacists’ attitudes toward participation in training.

*“We need formal, specific, and continuous training for the pharmacist regarding preparedness, because, you know, this field is ever growing, and always requires staff to be up-to-date” (FC1, P3).*

“HMC has a simulation center, and they do simulation training for physicians and nurses. I think providing the pharmacists with such real-life case scenarios would be a great idea.” (FC3, P2).

#### 5.1.2 Role identification

The duties and responsibilities of healthcare professionals including physicians and nurses during emergency disasters are clearly identified; however participants reported that there is still lack of clarity regarding pharmacist role. Hence, they recommended that policymakers clearly identify their exact roles to ensure that they know what is expected from them and that they can work with other healthcare providers efficiently and effectively.

*“So, one of the major points that I feel we need to have at the level of corporate and at the national level is to have a clear role for the pharmacist and the clinical pharmacist during the emergency disaster. We are equally sharing accountability and responsibility, with the nurses and doctors in managing the situation. So, our role should be very clear.” (FC1, P6).*

#### 5.1.3 Action plan

Participants emphasized the importance of having a pharmacy-based action plan to ensure that all the required tasks are properly implemented and maintained during emergencies. This action plan should encompass the exact roles of pharmacists, pathways to guide the workflow, the resources needed, and relevant timelines.

*“For us here in the ED, whenever we have a disaster, whether it is related to an event or related to the daily routine things, we develop our job action plan, so we know what we do but this is because we have the experience of doing this on our own. I feel this has to be standardized and has to be disseminated to all pharmacists.” (FC3, P3).*

#### 5.1.4 Authority

Participants suggested that pharmacists in emergency situations should be granted expanded authority and privilege to make decisions in the ED, particularly independent prescribing. This will enable them to better contribute to patient care, reduce the load on other healthcare professionals, and help the team achieve the best possible outcomes in the event of a disaster.

*“We need extra privileges for prescribing; for example, in cases of emergencies, the physicians and nurses are too busy taking care of the patient, so we do not need to lose more time. This will help us take care of patients and help physicians and nurses achieve good outcomes in such a very busy time “(FC3, P4).*

### 5.2 System-related recommendations

#### 5.2.1 Observation programs

Participants agreed that it is crucial to learn from the experience of previous similar events such as the FIFA World Cup Russia. Sending observation teams that include pharmacists could be used to gather information about the successes and failures of these events. This would ensure that any positive outcomes could be reproduced, and any mistakes could be avoided.

*“I recommend sending a team of pharmacists and pharmacy managers to observe other events to benefit from their experience.” (FC1, P4).*

## Discussion

The current study explored ED pharmacy workforce’s perspectives on their preparedness and potential roles during mass gathering events such as the FIFA World Cup Qatar 2022™. We identified five major themes: readiness and preparedness for the World Cup, reflection on experience with providing care during the World Cup, perceived facilitators of providing care during the World Cup, perceived barriers/challenges of providing care during the World Cup, and recommendations for improving the workflow for future mass gathering events.

Despite the training provided by HMC and the ED, our study participants had mixed opinions on their preparedness. In the first phase of this project, ED pharmacists and clinical pharmacists reported that although awareness level was high, readiness for emergency disasters that could occur during the World Cup was still moderate (26). A plausible explanation could be the lack of pharmacy-specific training that was reported by the participants. Regardless, the perceived preparedness was still higher than the international level which was found to be poor in a recent systematic review (21). This emphasizes the effectiveness of the preparedness measures implemented by HMC, the ED, and the pharmacy department that were highlighted in our study such as the practice governing protocols and pathways, language bank, staff deployment, and staff training particularly practice drills. Therefore, we encourage health authorities in countries that will be hosting mass gathering events in the future to consider incorporating the measures presented in this study with appropriate modification to suit the context of the hosting country and its healthcare system.

It is worth pointing out that the preparedness of healthcare practitioners including pharmacists for mass gathering events has not been explored before as previous studies focused on hospital preparedness rather than staff with no mention for the role of the pharmacy department (33–35). It is hence imperative to investigate the preparedness of healthcare professionals prior to the event, particularly those working in the ED. This will identify the areas of weaknesses which will allow policy makers to develop strategies and training opportunities to address the exact gaps in the workflow and in knowledge, which could subsequently enhance the response of the emergency medical services. Additionally, the role of the pharmacy department and pharmacy workforce should be thoroughly explored as this will enable the appropriate integration of pharmacists into the disaster response multidisciplinary team which could improve the care provided to patients while reducing the workload on other health practitioners.

Participants in our study perceived their overall experience of working and caring for patients during the World Cup as positive and successful. They also described it as smooth-flowing and less demanding than expected. This could be attributed to the comprehensive strategies that prepared the staff for worst-case scenarios (e.g., collapse of stadium). Nonetheless, no major incidents occurred during the tournament and hence this finding could have been less positive if the staff had experienced a disaster. Additionally, the pharmacy workforce stated that their previous experience in mass gathering events held in Qatar had a positive influence on their experience during the World Cup. This finding is in parallel with a cross-sectional, Europe-wide survey study which showed that pharmacists who experienced disasters were more likely to develop standard operating procedures for future events which made them more prepared for any emergency disaster (22).

Participants discussed several internal and external facilitators to the effective provision of care during mass gathering events. One of the most prominent facilitators was the mobile command units, dedicated crews, and mobile major incidents response units in each stadium and fan zone which significantly reduced the workload on the ED. This is in line with a recent study from India which revealed that allocating medical staff to the area of a religious mass gathering effectively contributed to the success of the event (36). Another key facilitator was the high public health awareness amongst the residents within the State of Qatar. The WHO advocated for health promotion and public information in mass gathering planning to ensure public health risks, health promotion and public information opportunities are identified and realized. It was also emphasized that organizers should engage communities and utilize the event to stimulate action to promote healthy behaviors in the local population before, during and after the event (37). In addition to the benefits presented by the WHO, findings from this study suggest that such interventions could also support the health workforce and reduce the workload on the emergency medical services which could ultimately enhance the response capabilities of the healthcare system. Thus, it is pivotal to implement health promotion interventions and public information campaigns prior to the event and to evaluate the efficacy of the implemented strategies in order to support the continuous quality improvement and to share learning to add value to the mass gathering medicine evidence.

Several barriers to the provision of care during mass gathering events have been reported in the current study. Pharmacists reported that they had to work for longer hours due to the expansion of services and the insufficiency of staff. A recently published study from Qatar showed that the prevalence of burnout syndrome amongst pharmacy professionals was relatively low (17–19%) compared to the global prevalence, however overtime working hours per month was independently associated with a higher risk of burnout (38). We encourage pharmacy directors in Qatar and in other countries that will host mass gathering events to allocate more staff to the ED during such high demand periods to reduce the occurrence of burnout and ensure the safety of patients.

Whilst participants described the institute formulary to be sufficient and comprehensive, issues related to medication availability arose during the World Cup. This was mainly related to chronic medications such as monoclonal antibodies; hence we advise that the FIFA in collaboration with the WHO and the hosting country formulate a list of essential medications to be shared with the event attenders to emphasize on the importance of bringing sufficient supply of these medications. It is noteworthy that barriers in our study were reported by the pharmacists and clinical pharmacists while supervisors were not aware of any barriers which indicates that the communication between staff and supervisors is poor. Accordingly, we strongly suggest that supervisors open several communication channels with the staff such as distributing anonymized surveys post event to encourage feedback about their experience which will guarantee the organizational learning-continuous improvement.

Several recommendations have been suggested by participants to improve the workflow of ED pharmacists in future mass gathering events in Qatar and across the globe. The primary recommendation was the pharmacist’s role identification in mass gathering and development of pharmacy action plan to outline the series of steps that pharmacists and clinical pharmacists should undertake within a realistic timescale. Similarly, previous literature highlighted the lack of clarity in regard to the exact roles and responsibilities of pharmacists during mass gatherings as well as the uncertainty around the pharmacist estimated demand (13, 18, 19). Additionally, participants suggested granting increased authority to pharmacists during mass gatherings. We therefore recommend that future research investigate the potential role for pharmacists in such events to shape their roles and propose action frameworks. This should be followed by an assessment of the feasibility and scalability of such roles in real-world settings. We also recommend utilizing theoretical frameworks in developing such interventions and services as they provide an in-depth understanding of the structural and psychological determinants of behavior at different levels (i.e., individual, interpersonal, organizational, community, and societal levels). This enables theoretically informed interventions to create a sustainable behavior change (39–41).

Participants also suggested improvements to the training provided prior to the event to enhance their preparedness. Providing simulation training was recommended by our participants alongside the continuous and mandatory training for pharmacists working under all facilities rather than the ED only to ensure their readiness to be reallocated to the ED. Multiple studies have recognized simulation training as a promising pedagogical tool to improve the learning outcomes and increase the pharmacy workforce preparedness for high-stress, high-impact clinical scenarios and medical emergencies (42–45). Nevertheless, the evidence underpinning this innovative teaching method is still conflicting and further research is required to determine its educational and clinical effectiveness (43, 44). We also encourage policy makers to conduct a learning needs assessment before offering educational and training initiatives as this will ensure that the sessions are tailored to the needs of the audience (46).

One of the strengths of this study is that it is part of a larger mixed methods study comprising quantitative and qualitative stages which allowed for the first time the exploration of the perspectives of the pharmacy workforce on the provision of pharmaceutical care during mass gathering events (26). Additionally, we promoted participants’ ability to freely express their views and enrich the discussion by ensuring confidentiality and anonymity, conducting a separate focus group for supervisors, and using a semi-structured topic guide.

Our study, on the other hand, has some limitations. First, participants might have been conservative in expressing their opinions in the presence of coworkers. Second, it is possible that only pharmacists with either very strong positive or very strong negative opinions toward their experience in mass gathering events might have participated, resulting in nonrespondent bias and hence raising the question of validity. Third, only one focus group for supervisors was conducted and the sample size was small due to the limited number of available participants.

## Conclusion

The current study revealed that despite perceived barriers, pharmacists reported positive views in relation to their experience in providing pharmaceutical care during mass gathering events. Participants also provided recommendations that should be considered to enhance the preparedness of pharmacists for such events and effectively integrate the pharmacy workforce in the disaster response multidisciplinary team. Future research should focus on the development of a framework for pharmacy departments to adopt during mass gathering events.

## Data availability statement

The original contributions presented in the study are included in the article/supplementary material, further inquiries can be directed to the corresponding author.

## Author contributions

LN: Conceptualization, Data curation, Formal analysis, Funding acquisition, Investigation, Methodology, Project administration, Resources, Software, Supervision, Validation, Visualization, Writing – original draft, Writing – review & editing. NI: Conceptualization, Data curation, Investigation, Methodology, Project administration, Validation, Writing – review & editing. SA: Data curation, Formal analysis, Investigation, Methodology, Software, Validation, Writing – original draft, Writing – review & editing. AP: Methodology, Supervision, Visualization, Writing – review & editing. SA: Project administration, Resources, Visualization, Writing – review & editing. MH: Resources, Supervision, Visualization, Writing – review & editing.

## References

[1] Communicable disease alert and response for mass gatherings. Key considerations. Geneva: World Health Organization (WHO) (2008).

[2] McCloskey B Zumla A Ippolito G Blumberg L Arbon P Cicero A. Mass gathering events and reducing further global spread of COVID-19: a political and public health dilemma. Lancet. (2020) 395:1096–9. doi: 10.1016/S0140-6736(20)30681-4, PMID: 32203693 PMC7138150

[3] Sabra JP Cabañas JG Bedolla J Borgmann S Hawley J Craven K. Medical support at a large-scale motorsports mass-gathering event: the inaugural formula one United States grand prix in Austin. Texas Prehospital Disaster Med. (2014) 29:392–8. doi: 10.1017/S1049023X14000636 25068212

[4] Koski A Kouvonen A Sumanen H. Preparedness for mass gatherings: factors to consider according to the rescue authorities. Int J Environ Res Public Health. (2020) 17:1361. doi: 10.3390/ijerph17041361, PMID: 32093217 PMC7068565

[5] Zeitz K Bolton S Dippy SSR Dowling Y Francis L Thorne J. Measuring emergency services workloads at mass gathering events. Aust J Emerg Manag. (2007) 22:23–30.

[6] Arbon P. Planning medical coverage for mass gatherings in Australia: what we currently know. J Emerg Nurs. (2005) 31:346–50. doi: 10.1016/j.jen.2005.03.002, PMID: 16126098

[7] Koski A Pappinen J Kouvonen A Nordquist H. Preparedness for mass gatherings: rescue and emergency medical services’ workloads during mass gathering events. Scand J Trauma Resusc Emerg Med. (2022) 30:15. doi: 10.1186/s13049-022-01003-7, PMID: 35248139 PMC8898448

[8] Fleischauer AT Gaines J. Enhancing surveillance for mass gatherings: the role of syndromic surveillance. Public Health Rep. (2017) 132:95S–8S. doi: 10.1177/0033354917706343 28692398 PMC5676502

[9] Emergencies: WHO's role in mass gatherings: World Health Organization (WHO). (2019). Available at: https://www.who.int/news-room/questions-and-answers/item/what-is-who-s-role-in-mass-gatherings (Accessed March 29, 2023).

[10] Our accreditations: Hamad Medical Corporation. (2023). Available at: https://www.hamad.qa/EN/About-Us/Our-Accreditations/Pages/default.aspx (Accessed January 8, 2023).

[11] Qatar Population (LIVE): Worldometers. (2023). Available at: https://www.worldometers.info/world-population/qatar-population/ (Accessed March 29, 2023).

[12] Climate and Average Weather Year Round in Doha, Qatar: Weather Spark. (2016). Available at: https://weatherspark.com/y/105083/Average-Weather-in-Doha-Qatar-Year-Round (Accessed March 29, 2023).

[13] Alomi Y. National Mass Gathering Pharmaceutical Care Program at MOH in Saudi Arabia. J Pharm Pract Commun Med. (2016) 2:102–3. doi: 10.5530/jppcm.2016.3.9

[14] González-Val R Marcén M. Mass gathering events and the spread of infectious diseases: evidence from the early growth phase of COVID-19. Econom Hum Biol. (2022) 46:101140. doi: 10.1016/j.ehb.2022.101140, PMID: 35525103 PMC9027297

[15] Ministry of Public Health, Qatar and WHO collaborating to implement public health security measures as FIFA World Cup approaches: World Health Organization (WHO) (2022). Available at: https://www.who.int/news-room/feature-stories/detail/ministry-of-public-health--qatar-and-who-collaborating-to-implement-public-health-security-measures-as-fifa-world-cup-approaches (Accessed March 29, 2023).

[16] HMC prepares pharmacists for FIFA World Cup in Qatar: Qatar Tribune (2022). Available at: https://www.qatar-tribune.com/article/110432/NATION/HMC-prepares-pharmacists-for-2022-FIFA-World-Cup-in-Qatar (Accessed January 8, 2023).

[17] Isleem N Shoshaa S AbuGhalyoun A Khatib M Naseralallah LM ibn-Mas'ud Danjuma M. Critical care tele-pharmacy services during COVID-19 pandemic: a qualitative exploration of healthcare practitioners' perceptions. J Clin Pharm Ther. (2022) 47:1591–9. doi: 10.1111/jcpt.13709, PMID: 35699243 PMC9350019

[18] Cecchi A Carchietti E. A planning model of pharmaceutical needs for mass gatherings at public special events. Pharm Regul Aff. (2013) 2:2–3. doi: 10.4172/2167-7689.1000107

[19] Alomi Y Alhennawi K Khayayt N. Pharmacy workload and workforce requirements at MOH hospitals during ten years mass gathering hajj (2006-2015) in Makkah region, Saudi Arabia. J Pharm Pract Commun Med. (2017) 3:s75–83. doi: 10.5530/jppcm.2017.4s.52

[20] Ford H Trent S Wickizer S. An assessment of state Board of Pharmacy Legal Documents for public health emergency preparedness. Am J Pharm Educ. (2016) 80:20. doi: 10.5688/ajpe80220, PMID: 27073273 PMC4827571

[21] McCourt E Singleton J Tippett V Nissen L. Disaster preparedness amongst pharmacists and pharmacy students: a systematic literature review. Int J Pharm Pract. (2021) 29:12–20. doi: 10.1111/ijpp.12669, PMID: 32881173

[22] Schumacher L Bonnabry P Widmer N. Emergency and disaster preparedness of European hospital pharmacists: a survey. Disaster Med Public Health Prep. (2021) 15:25–33. doi: 10.1017/dmp.2019.112, PMID: 31739816

[23] Al-Ziftawi NH Elamin FM Mohamed Ibrahim MI. Assessment of knowledge, attitudes, and readiness to practice regarding disaster medicine and preparedness among university health students. Disaster Med Public Health Prep. (2021) 15:316–24. doi: 10.1017/dmp.2019.157, PMID: 32115009

[24] Ahmad Suleiman M Magaji MG Mohammed S. Evaluation of pharmacists' knowledge in emergency preparedness and disaster management. Int J Pharm Pract. (2022) 30:348–53. doi: 10.1093/ijpp/riac049, PMID: 35781567 PMC9384291

[25] Aljabri A Bakhsh H Baageel A al-Nimari S Alshehri S Bakadam B. Hospital pharmacy preparedness and pharmacist role during disaster in Saudi Arabia. Risk Manag Health Policy. (2021) Volume 14:5039–46. doi: 10.2147/RMHP.S343789, PMID: 34955660 PMC8694794

[26] Isleem N Naseralallah L Koraysh S Abu Ghalyoun A Alnaimi S Pallivalapila A. Disaster preparedness amongst emergency pharmacists for the FIFA world cup Qatar 2022™: a cross-sectional survey. Risk Manag Health Policy. (2023) Volume 16:573–83. doi: 10.2147/RMHP.S404367, PMID: 37038373 PMC10082576

[27] Kitzinger J. Qualitative research: introducing focus groups. Bmj. (1995) 311:299–302. doi: 10.1136/bmj.311.7000.299 7633241 PMC2550365

[28] Stewart DSP Rook D. Focus groups: theory and practice. 3rd ed. Los Angeles: SAGE Publications (2014).

[29] Tong A Sainsbury P Craig J. Consolidated criteria for reporting qualitative research (COREQ): a 32-item checklist for interviews and focus groups. Int J Qual Health Care. (2007) 19:349–57. doi: 10.1093/intqhc/mzm042, PMID: 17872937

[30] Onwuegbuzie AJ Leech NL. Generalization practices in qualitative research: a mixed methods case study. Qual Quant. (2010) 44:881–92. doi: 10.1007/s11135-009-9241-z

[31] Barbour R. Introducing qualitative research: a student’s guide. 2nd ed. London: SAGE Publications (2014).

[32] Braun V Clarke V. Using thematic analysis in psychology. Qual Res Psychol. (2006) 3:77–101. doi: 10.1191/1478088706qp063oa

[33] Betlehem J Schaefer J. Emergency medical preparedness during the 2006 world cup in Frankfurt, Germany. Disasters. (2010) 34:155–63. doi: 10.1111/j.1467-7717.2009.01119.x, PMID: 19682004

[34] Valesky W Silverberg M Gillett B Roblin P Adelaine J Wallis LA. Assessment of hospital disaster preparedness for the 2010 FIFA world cup using an internet-based, long-distance tabletop drill. Prehosp Disaster Med. (2011) 26:192–5. doi: 10.1017/S1049023X11006443, PMID: 22107770

[35] al-Romaihi H al-Dahshan A Kehyayan V Shawky S al-Masri H Mahadoon L. Knowledge, attitude, and training of health-care workers and preparedness of hospital emergency departments for the threat of communicable diseases at mass gathering events in Qatar: a cross-sectional study. Disaster Med Public Health Prep. (2023) 17:e49. doi: 10.1017/dmp.2021.296, PMID: 34668848

[36] Goel, MD K Sharma, MD N Bhogal, MHA RS Singh, MD A. Management of a religious mass gathering in North India: Parkash Utsav 550. J Emerg Manag. (2021) 19:379–85. doi: 10.5055/jem.0627, PMID: 34580853

[37] World Health Organization (WHO). Public health for mass gatherings: key considerations. (2015). Available from: https://www.who.int/publications/i/item/public-health-for-mass-gatherings-key-considerations

[38] Eltorki Y Abdallah O Riaz S Mahmoud S Saad M Ez-Eldeen N. Burnout among pharmacy professionals in Qatar: a cross-sectional study. PLoS One. (2022) 17:e0267438. doi: 10.1371/journal.pone.0267438, PMID: 35511925 PMC9071121

[39] Naseralallah L Stewart D Azfar Ali R Paudyal V. An umbrella review of systematic reviews on contributory factors to medication errors in health-care settings. Expert Opin Drug Saf. (2022) 21:1379–99. doi: 10.1080/14740338.2022.2147921, PMID: 36408597

[40] Reason J. Human error: models and management. BMJ. (2000) 320:768–70. doi: 10.1136/bmj.320.7237.768, PMID: 10720363 PMC1117770

[41] Skivington K Matthews L Simpson SA Craig P Baird J Blazeby JM. A new framework for developing and evaluating complex interventions: update of Medical Research Council guidance. BMJ. (2021) 374:n2061. doi: 10.1136/bmj.n2061 34593508 PMC8482308

[42] Resende K Cavaco A Luna-Leite M Acacio B Pinto N Neta MD. Training and standardization of simulated patients for multicentre studies in clinical pharmacy education. Pharm Pract. (2020) 18:2038. doi: 10.18549/PharmPract.2020.4.2038, PMID: 33224323 PMC7672484

[43] Garnier A Vanherp R Bonnabry P Bouchoud L. Use of simulation for education in hospital pharmaceutical technologies: a systematic review. Eur J Hosp Pharm. (2023) 30:70–6. doi: 10.1136/ejhpharm-2021-003034, PMID: 34949651 PMC9986932

[44] Aura S Sormunen M Jordan S Tossavainen K Turunen H. Learning outcomes associated with patient simulation method in pharmacotherapy education: an integrative review. Simul Healthc. (2015) 10:170–7. doi: 10.1097/SIH.0000000000000084, PMID: 25932709

[45] Thompson Bastin ML Cook AM Flannery AH. Use of simulation training to prepare pharmacy residents for medical emergencies. Am J Health Syst Pharm. (2017) 74:424–9. doi: 10.2146/ajhp160129, PMID: 28274986

[46] Al-Ismail MS Naseralallah LM Hussain TA Stewart D Alkhiyami D Rasheed HMA. Learning needs assessments in continuing professional development: a scoping review. Med Teach. (2022) 1-9 10.1080/0142159X.2022.2126756 36179760

